# Effect of Polyacrylonitrile Precursor Orientation on the Structures and Properties of Thermally Stabilized Carbon Fiber

**DOI:** 10.3390/ma14123237

**Published:** 2021-06-11

**Authors:** Bin Wang, Chenggao Li, Weiyu Cao

**Affiliations:** 1 Central Research Institute of Building and Construction Co., Ltd., MCC, Beijing 100088, China; mccwangbin@126.com; 2 Key Lab of Structures Dynamic Behavior and Control, Harbin Institute of Technology, Ministry of Education, Harbin 150090, China; 3 Key Lab of Smart Prevention and Mitigation of Civil Engineering Disasters of the Ministry of Industry and Information Technology, Harbin Institute of Technology, Harbin 150090, China; 4 Harbin Institute of Technology, School of Civil Engineering, Harbin 150090, China; 5 State Key Laboratory of Organic-Inorganic Composites, Beijing University of Chemical Technology, Beijing 100029, China; 6 The Key Laboratory of Education Ministry on Carbon Fiber and Functional Polymer, Beijing University of Chemical Technology, Beijing 100029, China

**Keywords:** PAN precursor, carbon fiber, orientation degree, thermal stabilization, structures and properties

## Abstract

The thermal stabilization process of polyacrylonitrile (PAN) precursor fiber was the key step to prepare high-performance carbon fiber. During the thermal stabilization process, the aggregation structure and the reactivity of molecular chains have significant effects on the microstructures and mechanical properties of carbon fiber. In the present paper, the effects of the orientation structure of PAN precursor fiber on the thermal stabilization reaction and the mechanical properties of carbon fiber were experimentally studied. Using multi-dimensional structural and mechanical properties tests, such as XRD, DSC, ^13^C NMR and Instron machine testing, the crystalline and skeleton structure, exothermic behavior, and tensile properties of PAN precursor fiber with different orientations in the process of thermal stabilization were characterized to reveal the relationship between microstructure evolution and tensile properties. The results showed that the orientation structure of PAN precursor fiber had an obvious effect on the thermal stabilization process and the tensile stress–strain characteristic. When the heat treatment temperature was lower than 200 °C, the crystallinity and crystallite size of PAN fibers with higher orientation degrees increased significantly. After sufficient thermal stabilization, the original PAN precursor fiber with a higher orientation degree could form more aromatic lamellar structures and showed better regularity. Furthermore, the yield strength and initial modulus of the fibers with a higher orientation degree increased due to the formation of more aromatic rings. The maximum increase in the percentages of yield strength and tensile modulus of the PAN fibers were achieved when the heat-treated temperature was 200 °C, and the percentage values were 138.4% and 158.7% compared to the precursor without heat-treatment. In addition, the elongation at break of the fibers with a higher orientation degree was also relatively larger.

## 1. Introduction

PAN-based carbon fiber has excellent mechanical properties, as well as fatigue and corrosion resistances. In recent years, its application field has been extended rapidly to construction, energy, transportation, automation, and other fields. Aiming at different application requirements, the development of high-performance carbon fiber is significant. Furthermore, how to match the structure and performance of carbon fiber needs to be investigated to improve the mechanical and fatigue properties of carbon fiber.

Previous studies have found that, for carbon fiber with more than 92% carbon content, the size and orientation of graphite microcrystalline, graphite lamellar spacing, and other structural parameters have a significant effect on the mechanical properties of the carbon fiber [1,2]. In addition to reasonable control of growth perfection and preferred orientation structure of graphite microcrystals during the carbonization process [3,4], it is also an important step to control the characteristic structure of PAN fibers in the process of precursor preparation and thermal stabilization. Especially in the process of thermal stabilization, the linear molecular chains of PAN fiber convert into a heat-resistant ladder structure through a series of chemical reactions and physical changes. The basic ladder structure decides the micro and macro properties of carbon fiber through the carbonization process. For a controllable preparation process of high-performance carbon fiber, it is of great significance to study the chemical reactions and structural evolution during the thermal stabilization process of PAN fiber.

Some researchers have focused on the chemical reactions of the PAN molecular chain in thermal stabilization, which led to structural changes, including the mechanism of cyclization, dehydrogenation and oxidation [5,6], chemical reaction rate [7,8], the interaction among the three kinds of chemical reactions [9,10,11], and the characteristics of the trapezoidal cross-linking structure [12,13,14]. However, the copolymerization components, heat treatment time, temperature, and atmosphere also have complex effects on the chemical reaction structure [15,16,17,18]. At present, there is no unified conclusion about the chemical structure of thermally stabilized fibers.

Some researchers focused on the change in the aggregate structure and its influence on the thermal stabilization reaction [19,20]. Ko et al. [21,22,23] found that the transition from a PAN linear chain structure unit to a trapezoidal structure begins in the fiber’s amorphous region and then enters the crystal region at a high temperature. Liu et al. [24,25] further studied the orientation structure changes of PAN fiber during the thermal stabilization process. It was found that the disordered region of the PAN fiber molecular chain firstly occurs in the chemical reaction at a lower heat treatment temperature, and the orientation degree of the crystal region did not change much at that time. With an increase of heat treatment temperature, the chemical reaction occurred in the ordered region, and the orientation of the crystal region decreased sharply. Considering the change in the orientation structure, it was considered that, on the one hand, the change in the orientation structure was affected by the mobility of the molecular segments. The aggregation structure could affect the mobility of the PAN molecular chain, and then affect the cyclization activity of the cyano group on the side of the PAN molecular chain [26]. On the other hand, the thermal stabilization reaction affected the evolution of the fiber orientation structure and the aggregation structure [27,28]. For the fiber orientation structure, it has not been reported whether the cross-linked trapezoidal structure based on the original ordered structure has certain characteristics and will further affect the growth and perfection of the ordered structure in the carbonization process, which plays a significant role in the mechanical properties of carbon fiber.

In the present study, PAN precursor fiber with different orientation structures was self-made using wet spinning. The effects of the orientation degree on the thermal stabilization reaction and mechanical properties of the fiber were studied by means of multi-dimensional structural characterization and mechanical properties tests, such as XRD, DSC, ^13^C NMR, and Instron machine testing. The effect mechanism of the orientation structure of the PAN precursor on the thermal stabilization reaction was revealed, which will provide a theoretical basis for the improvement of the mechanical properties of PAN-based carbon fiber.

## 2. Materials and Methods

### 2.1. Raw Materials and Sample Preparation

In the present paper, four kinds of PAN precursor fibers with different orientation structures, containing IA copolymer (≤1%), were produced by changing the stretching ratios during wet spinning. Furthermore, the aggregation structure parameters of the above precursor samples are shown in Table 1.

The total orientation degree of the molecular chains in the PAN precursor fiber was determined based on a sound velocity meter produced by the Chinese Academy of Sciences. The total orientation degree and the orientation degree of the amorphous region are calculated using Equation (1) [29] and Equation (2) [30], as follows:

(1) $$f_{sum}=1-\frac{C_{u}^{2}}{C_{x}^{2}}\times 100$$

where *f_sum_* represents the total orientation degree of the molecular chains in the PAN fiber. *C_u_* is a constant of the speed of sound (2.1 km/s) for PAN fibers of non-orientated samples. *C_x_* is the measured sound velocity for PAN samples with the orientation degree.

(2) $$f_{amo}=\frac{(E_{sum}-E_{u})-X_{cry}\cdot f_{cry}\cdot E_{sum}}{E_{sum}(1-X_{cry})}$$

where *f_amo_* represents the orientation degree of the amorphous region in the PAN fiber. *E_u_* is a constant of the velocity modulus (11.3*C_u_*^2^), and *E_sum_* is the measured velocity modulus for PAN fiber (11.3*C_x_*^2^). *X_cry_* and *f_cry_* represent the crystallinity of the PAN fiber and the orientation degree of the crystalline region, respectively.

(3) $$f_{cry}=\frac{360-\sum H_{i}}{360}\times 100\%$$

where *f_cry_* is the FWHM of the *i*th peak in the azimuthal scanning X-ray diffraction pattern [31].

The thermally stabilized PAN fiber was prepared using the continuous peroxidation process. The temperature range was from 190 °C to 260 °C. The temperature of each stage was 190 °C, 200 °C, 230 °C, 250 °C, and 260 °C, and the peroxidation staying time of each temperature was 10 min.

### 2.2. WARD Tests

The crystalline structure measurements of the PAN fiber were performed by heating the sample on a hot stage (Anton Paar TTK 450 low temperature control, Anton Paar TCU 100 (Graz, Austria) temperature control device and medium and low temperature accessories) and exposing the sample to an X-ray diffractometer (X’ Pert PRO MPD X-ray diffractometer of PANalytical, (Almelo, The Netherlands)). Kα radiation of Cu target (*λ* = 0.15418 nm), tube voltage (40 kV), tube current (40 mA) and Ni filter were applied. A super energy array probe (X’Celerator) was selected with a scanning step width of 0.033° and scanning range of 5–50° in air atmosphere with a heating rate 10 °C/min, from 50 to 300 °C. The data were collected at intervals of 10 °C lasting for 10 min. The crystallinity and crystallite size of the PAN fiber were calculated using Equation (4) [32] and Equation (5) [33], as follows.

(4) $$X_{cry}=\frac{S_{cry}}{S_{amo}+S_{cry}}\times 100\%$$

where *X_cry_* represents the crystallinity of the PAN fiber, *S_cry_* and *S_amo_* are the integrated intensities of the peaks for the crystalline component and amorphous phase, respectively.

(5) $$L_{\left(a\right)}=\frac{K\lambda }{\beta \cos\theta }$$

where *L_a_* represents the crystallite size of the 100 plane for the PAN fiber, *β* is the half-width of the 100 diffraction peak, *K* is a constant (0.89), *λ* is the wavelength of the Cu Kα radiation and *θ* is the Bragg angle of the 100 diffraction peak.

### 2.3. C-NMR Tests

^13^C-NMR (Bruker AV-300 (Bruker, Switzerland) solid state high resolution nuclear magnetic resonance) was used to characterize the skeleton structure of the PAN precursor during the thermal stabilization process. The resonance frequency was 73.5 MHz and the pulse width was 6.6 μs. The cumulative scanning time was from 300 s to 3175 s, the cyclic delay time was 5 s and the contact time was 3 ms. A CP/MAS probe with a diameter of 4 mm was used in the experiment, and the rotor rotation rate was 8.5 kHz.

### 2.4. DSC Tests

The exothermic behavior of the PAN precursor fiber with different orientation structures during the thermal stabilization process was measured by differential scanning calorimetry (DSC, TA instrument Q100 (New Castle, DE, USA)). The thermal treatment process was carried out at a heating rate of 10 °C/min, from 50 to 400 °C in air atmosphere.

### 2.5. Tensile Properties

The tensile properties of PAN monofilament, before and after thermal stabilization, were tested by the universal material testing machine (SHUMADZU AG-1S (Kyoto, Japan)). The length of the monofilament was 20 mm, the stretching speed was 2 mm/min. The average values of tensile properties for each condition were obtained through the testing of 25 samples. The characteristic mechanical property parameters were calculated according to the tensile stress–strain curves.

## 3. Results and Discussion

### 3.1. Effect of Orientation Structure of PAN Fiber on the Aggregation Structure during the Thermal Stabilization Reaction

**Figure 1:**
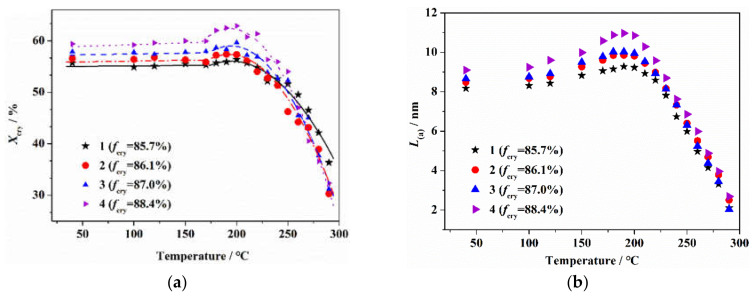
Crystalline structure for PAN fibers after heat treatment at different temperatures lasting for 10 min: (**a**) crystallinity and (**b**) crystallite size.

Figure 1 shows the crystalline structure of PAN fibers after the heat treatment at different temperatures, lasting for 10 min. As seen, with the increase in the heat treatment temperature, the crystallinity and crystallite size of the PAN fiber first increased to the maximum at 200 °C, and then decreased sharply. Before 200 °C, the growths of crystallinity and crystallite size of original fibers with higher orientations were relatively larger. When the heat treatment temperature was higher than 200 °C, the decreases in crystallinity and crystallite size of the original fibers with higher orientations were relatively sharp. For heat treatment at 250 °C, the crystalline structure was completely deconstructed, and there were no obvious differences in the crystalline structure of fibers with different orientations. This indicated that with the increase in the heat treatment temperature, the effect mechanism of the orientation structure of the PAN fiber on the aggregate structure was different.

Previous studies have found that an initial thermal stabilization reaction occurred in the amorphous region of the PAN molecular chains [27]. It was considered that, with the increase in heat treatment temperature before reaching 200 °C, the cyclization reaction of the molecular chains in the amorphous region resulted in the shrinkage of the linear molecular chains of PAN which gradually transformed into a ring structure, which increased the internal stress of the system. At that time, the more regular packing molecular chains in the pseudo-crystalline region were easily able to self-rearrange under the internal stress, which promoted the formation of the crystalline structure and the increases in crystallinity and grain size. As is known, fibers with a higher orientation structure have more regular molecular segments, which were more easily able to form crystalline regions under the induction of cyclization stress which resulted in the increase in crystallinity and crystallite size. With a further increase in the heat treatment temperature, the thermal stabilization reaction gradually evolved into the crystalline region of the PAN precursor [27]. For heat treatment at 200 °C, the molecular chains with imperfect structures in the crystalline region began to gradually cyclize. In the process of the formation of cross-linked ladder structure of the PAN molecular chain, the long chain structure with a regular orientation in the original crystal region was gradually destroyed, which led to a decrease in the crystallinity and crystallite size. With the increase in the heat treatment temperature, the thermal stabilization reaction of the molecular chains in crystalline region was accelerated, which led to the original crystalline region splitting into some smaller microcrystals, and the crystallinity and crystallite size of the fiber further decreased. For fibers with a higher degree of orientation, the segment arrangement in the crystalline region was more regular, and the cyclization reaction was more rapid over a short time, which obviously decreased the crystallinity and crystallite size. For heat treatment at 250 °C, the thermal stabilization reaction occurred in the crystalline region of the fiber, and the crystalline structure was completely deconstructed.

### 3.2. Effect of Orientation Structure of PAN Fiber on the Characteristic Structure during the Thermal Stabilization

**Figure 2:**
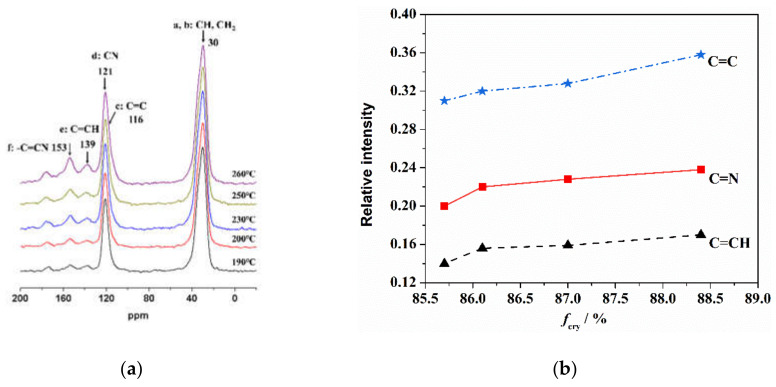
Typical structure of thermally stabilized PAN fiber determined by ^13^C-NMR at different temperatures lasting for 10 min of (**a**) characteristics peak and (**b**) orientation at 260 °C.

Solid state ^13^C-NMR was used to characterize the existing state of the carbon atoms in the skeleton chain of the PAN fiber during the thermal stabilization reaction. The influence of the orientation structure of the PAN fiber on the thermal stabilization reaction was analyzed and is shown in Figure 2. As seen in Figure 2a, CH and CH_2_ (at 30 ppm) were the intrinsic carbon atom structures of the PAN molecular chain, and –C≡N (at 121 ppm) was the carbon atom structure of the cyano group on the side of the PAN molecular chain. After the dehydrogenation, the intrinsic carbon atom structures mainly changed to C=C (at 116 ppm) without hydrogen and C=CH (at 139 ppm) with only one hydrogen. In addition, after the cyclization of side nitriles, the intrinsic carbon atom structures of -C≡N changed to C=N (at 153 ppm). With the increase in the heat treatment temperature, the cyclization and dehydrogenation reactions gradually progressed, and the characteristic peaks of the C=C, C=CH and C=N structures gradually increased.

**Figure 3:**
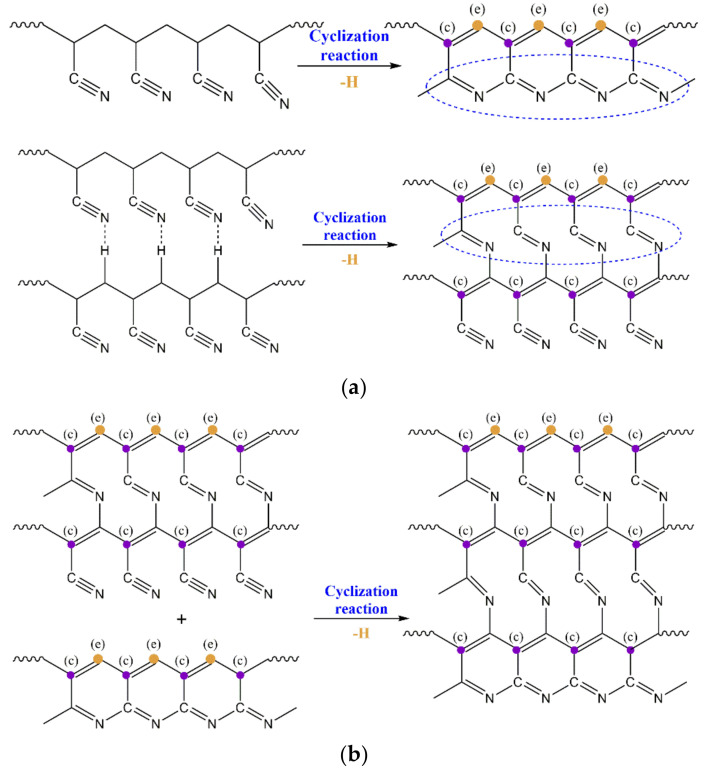
Typical structure evolution of PAN fiber during the thermal stabilization of (**a**) initial stage of thermal stabilization (<200 °C) and (**b**) middle and later stages of thermal stabilization (200–260 °C).

The relative contents of the three characteristic carbon structures (C=CH, C=N and C=C) generated in the thermal stabilization process were calculated through the ratio of peak strength of C=CH, C=N and C=C divided by CH. As shown in Figure 2b, more C=N, C=CH and C=C functional groups formed in the fibers with a higher orientation after thermal stabilization. With the increase of orientation degree, the growth rate of the C=C structure was more obvious than that of the other two kinds of structures. This indicated that the orientation structure could promote the formation of a characteristic structure during the thermal stabilization reaction. As shown in Figure 3, in the initial stage of thermal stabilization (<200 °C), the cyclization occurred at the torsion of −CN, and the dehydrogenation of cyclization structure occurred gradually. This resulted in the content of C=N, C=CH and C=C increasing. As the adjacent −CN in the same PAN molecular chain repelled each other, and the hydrogen bonds between the adjacent CN and α-hydrogen atoms of neighboring chains formed and attracted each other, which led to the cyclization reaction in the molecular chains being more difficult. Compared with the disordered region structure, the relatively regular orientation structure was more conducive to the sufficient cyclization reaction between the molecular chains. As a result, the content of the C=N characteristic structure increased in the fibers with a higher degree of orientation. Furthermore, more intermolecular cross-linking structures could lead to the formation of more C=C characteristic structures than that of C=CH. In the middle and later stages of thermal stabilization (200–260 °C), further intermolecular cross-linking reactions formed based on the cyclized molecular chain, which was accompanied by the removal of small molecules, leading to the formation of a larger aromatic-ring-like ladder structure. This caused an increase of the relative content of the C=N and C=CH structures, and the relative content of the C=C structure increased rapidly and was higher than the other two kinds of characteristic structures. For the fibers with a higher orientation, the inter-chain cyclization and dehydrogenation structures were more ordered, which helped to promote the further cross-linking of the molecular chains and formed a larger area of aromatic lamellar structures.

### 3.3. Effect of Orientation Structure of PAN Fiber on the Exothermic Behavior during the Thermal Stabilization

**Figure 4:**
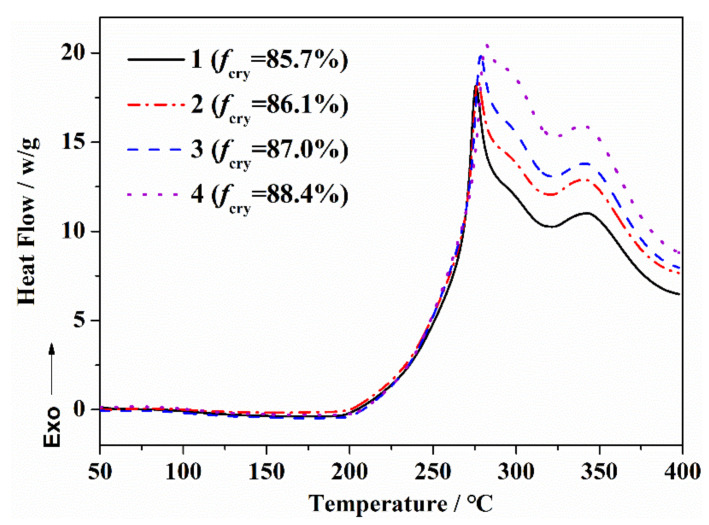
DSC curves of PAN fibers with different orientation structures during the thermal stabilization reaction.

The exothermic curves of the PAN fibers with different orientation degrees during the thermal stabilization reaction are shown in Figure 4, and the characteristic parameters of the exothermic curves are shown in Table 2. For the PAN fibers with a larger orientation degree, the initial temperature (T_onset_) during the thermal stabilization process moved towards a high temperature. After that, with the increase in the heat treatment temperature, the exothermic peak gradually became sharper, and the enthalpy (ΔH) was somewhat larger. The exothermic curves of PAN fibers with different orientations reflected the differences in the thermal stabilization reaction process. At lower temperatures, the molecular segments of PAN were difficult to move, and the thermal stabilization reaction first occurred in–CN, where the amorphous regions were close to each other. With the increase in the orientation degree, the less-disordered molecular chains in the amorphous region inhibited the thermal stabilization reaction process. With the increase in the heat treatment temperature, the mobility of the molecular segments increased, and the thermal stabilization reaction was further extended to the orientation region. The relatively regular orientation structure was helpful to generate more characteristic structures in a short time, which promoted the formation of the ladder aromatic ring and the increase in enthalpy (ΔH) of the reaction.

### 3.4. Effect of Orientation Structure of PAN Fiber on Stress–Strain during the Thermal Stabilization

**Figure 5:**
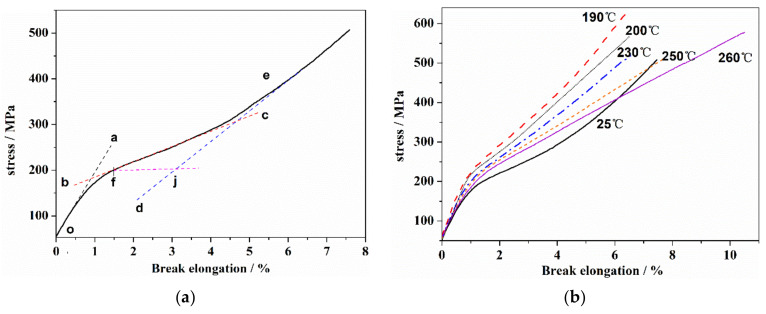
The stress–strain curves of PAN fiber. (**a**) PAN precursor fiber and (**b**) PAN fiber after the heat treatment.

The structure evolution of the PAN fiber during thermal stabilization had an obvious effect on the mechanical properties. The stress–strain curves of PAN fiber were shown in Figure 5. As seen in Figure 5a, the stress–strain curve of the PAN precursor had the typical structural characteristics of a linear polymer chain. At the initial stretching stage, the stress increase was in proportion to the increase in elongation. The slope of the oa segment (Figure 5a) was used to obtain the tensile modulus, E, which reflected the general elastic deformation ability caused by the bond length and the bond angle in the linear molecular chain under the external force. With the further increase in elongation, the PAN molecular segments were forced to move until the yield point, f, resulting in the high elastic deformation stage (bc segment). After that, with the increase in stress, the molecular chains began to slip, which showed obvious strain hardening phenomenon (de-segment). Subsequently, the stress continues to increase, and the molecular chains in some stress concentration areas gradually break, resulting in the overall fracturing of the sample. Figure 5b showed the stress–strain curves of the PAN fiber at different thermal stabilization stages. In addition, several typical mechanical characteristic parameters are shown in Table 3. As can be seen, with the increase in the heat-treated temperature, the yield strength and tensile modulus of the PAN fiber first increased, and then decreased rapidly when the temperature exceeded 200 °C. In comparison, the elongation at break decreased to the lowest value at 200 °C, after which an increased was observed. In addition, it can be observed that with the increase in temperature, the high elastic behavior gradually decreased until its complete disappearance at 200 °C.

**Figure 6:**
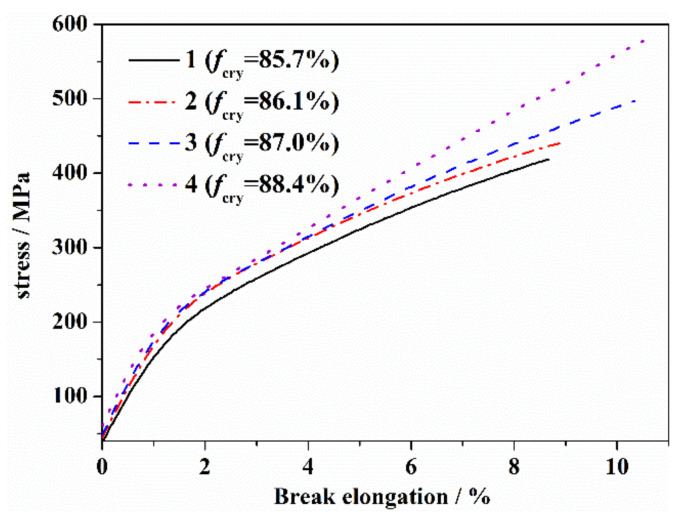
Stress–strain curves of PAN fibers with different orientations at a heat treatment of 260 °C.

The stress–strain curves of the PAN fibers with different orientations after heat treatment from 190 °C to 260 °C are shown in Figure 6, and several typical mechanical parameters are shown in Table 4. As is shown, the yield strength, tensile modulus and elongation at break of the thermal stabilized fibers were relatively larger, with the larger orientations. It was considered that the stress–strain curves of the PAN fiber reflected the evolution of fiber structure during the heat treatment. As a pseudo-crystalline polymer, the yield strength of the PAN fiber was mainly dependent on the crystalline structure. Before reaching 200 °C, the thermal stabilization reaction of the PAN fiber mainly stayed in the amorphous region and improved the bearing capacity of the fiber. At the same time, the crystallization generated from the cyclization promoted a slight increase in crystallinity, which led to an increase of the yield strength of the fiber. The change of the tensile modulus of the PAN fiber was similar to that of the yield strength. It was considered that the restriction of the crystalline structure in terms of the movement of molecular segments was the main factor to provide the resistance for the fiber to external force. With the increase in the heat treatment temperature, the increase of cross-linking of molecular chains and crystalline structure resulted in the maximum increase of the initial modulus of the fiber at 200 °C. After 200 °C, the thermal stabilization reaction extended to the molecular chains in the crystalline region. With the increase in the heat treatment temperature, the original crystalline structure was gradually destroyed, which led to a decrease in the yield strength and the tensile modulus. The elongation at break of the PAN fiber reflected the transition from linear long-chain molecules to a cross-linked structure. With the increase in the heat treatment temperature before reaching 200 °C, the degree of cyclization and cross-linking increased, and the system deformation was difficult and the elongation at break gradually decreased. After 230 °C, the crystalline structure of the fiber decomposed rapidly, and the effect of crystalline region on the movement of molecular segments decreased significantly. At the same time, a aromatic lamellar structure gradually formed, and the movement of the molecular chains of the fiber gradually changed into a deformation of the aromatic structure and the slip between the aromatic structures. With a further increasing of the temperature, the aromatic structure increased, which could produce a greater stretching deformation. After 250 °C, the elongation at break of the fiber was obviously higher than that of the precursor fiber.

The above discussion shows that the yield strength and initial modulus of the fibers with a higher orientation degree increased due to the formation of more aromatic rings after thermal stabilization. In addition, the elongation at break of the fibers with a higher orientation degree was also relatively larger after thermal stabilization. This was because the thermally stabilized fibers with an initial higher orientation degree had more aromatic structures, a larger aromatic ring area and better structural regularity. This led to a better axial ductility of the PAN fiber under external force.

## 4. Conclusions

In the present paper, PAN precursor fiber with different orientation structures was self-made via wet spinning. The effects of the orientation structure of the PAN precursor on the thermal stabilization reaction and mechanical properties were experimentally studied by means of multi-dimensional structural characterization and mechanical properties tests. The effect mechanism of the orientation structure of the PAN precursor on the thermal stabilization reaction was revealed, which will provide a theoretical basis for the improvement of mechanical properties of PAN-based carbon fiber. The following conclusions can be drawn:

1. The orientation structure of PAN fiber has an obvious effect on the aggregation structure during thermal stabilization. When the heat treatment temperature was lower than 200 °C, the crystallinity and crystallite size of the PAN fibers with a higher orientation degree increased significantly. When the heat treatment temperature was higher than 200 °C, the crystallinity and crystallite size of the fibers decreased sharply. In addition, the crystalline structure was completely deconstructed for heat treatment at 250 °C.
2. For fibers with a higher orientation, the inter-chain cyclization and dehydrogenation structure were more ordered after heat treatment, which helped to promote the further cross-linking of molecular chains and form a larger area of aromatic lamellar structures. Furthermore, the enthalpy (ΔH) of the reaction was observed to increase obviously.
3. The yield strength and initial modulus of the fibers with a higher orientation degree increased due to the formation of more aromatic structures, a larger aromatic ring area and better structures after thermal stabilization. In addition, the elongation at break of the fibers with a higher orientation degree was also relatively larger.
4. In the actual preparation process, in order to control the morphology of the PAN fiber, the tension drawing process is usually used in the process of thermal stabilization. Follow-up work will focus on the effect of tension drawing on the fiber orientation structure and thermal stabilization reaction in the process of thermal stabilization. It will further explore the effect of the thermal stabilization drafting behavior on the structure and mechanical properties of carbon fiber, and reveal the influence mechanism of stretching on the structure and properties of PAN carbon fiber during thermal stabilization processes.

## Figures and Tables

**Table 1:** The orientation structure parameters of PAN precursor fibers produced in the laboratory.

Sample Number	*F_sum_* (%)	*F_cry_* (%)	*F_amo_* (%)
1	$67.2_{-0.4}^{+0.3}$	$85.7_{-0.3}^{+0.2}$	$43.9_{-0.2}^{+0.1}$
2	$68.3_{-0.3}^{+0.5}$	$86.1_{-0.1}^{+0.1}$	$45.1_{-0.3}^{+0.2}$
3	$70.3_{-0.3}^{+0.4}$	$87.0_{-0.2}^{+0.3}$	$47.3_{-0.3}^{+0.3}$
4	$74.0_{-0.5}^{+0.5}$	$88.4_{-0.2}^{+0.2}$	$52.9_{-0.4}^{+0.5}$

Note: *f_sum_* is total orientation degree of the molecular chains; *f_cry_* is orientation degree of the crystalline region; *f_amo_* is the orientation degree of the amorphous region.

**Table 2:** Characteristic parameters of DSC curves of PAN fibers with different orientation structures during the thermal stabilization reaction.

Sample Number	*F_cry_* /%	T_onset_ /°C	T_p_ /°C	ΔH /J/g
1	85.7	197.3	275.3	2061
2	86.1	199.5	276.7	2089
3	87.0	200.0	278.5	2308
4	88.4	203.3	281.4	2603

**Table 3:** Mechanical properties of PAN fibers during thermal stabilization.

Heat Treatment Temperature (°C)	Yield Strength (MPa)	Tensile Modulus (GPa)	Elongation at Break (%)
Precursor	125	14.3	7.40
190	160	20.6	7.25
200	173	22.7	6.70
230	144	20.8	7.15
250	140	19.3	7.95
260	125	17.7	8.70

**Table 4:** Mechanical properties of thermally stabilized fibers with different orientations.

Sample	*f_cry_* (%)	Yield Strength (MPa)	Tensile Modulus (GPa)	Elongation at Break (%)	Tensile Strength (MPa)
1	$85.7_{-0.3}^{+0.2}$	$106_{-4}^{+2}$	$17.2_{-0.1}^{+0.2}$	$8.6_{-0.2}^{+0.2}$	$420_{-5}^{+4}$
2	$86.1_{-0.1}^{+0.1}$	$123_{-3}^{+3}$	$17.6_{-0.1}^{+0.2}$	$8.8_{-0.2}^{+0.2}$	$430_{-6}^{+5}$
3	$87.0_{-0.2}^{+0.3}$	$130_{-2}^{+4}$	$18.0_{-0.2}^{+0.2}$	$10.3_{-0.1}^{+0.3}$	$470_{-4}^{+4}$
4	$88.4_{-0.2}^{+0.2}$	$135_{-2}^{+3}$	$19.9_{-0.2}^{+0.1}$	$10.7_{-0.1}^{+0.2}$	$500_{-2}^{+3}$

## Data Availability

Data contain within the article.
